# Effect of the Strawberry Genotype, Cultivation and Processing on the Fra a 1 Allergen Content

**DOI:** 10.3390/nu10070857

**Published:** 2018-07-02

**Authors:** Elisabeth Kurze, Vanessa Kock, Roberto Lo Scalzo, Klaus Olbricht, Wilfried Schwab

**Affiliations:** 1 Biotechnology of Natural Products, Technische Universität München, Liesel-Beckmann-Str.1, 85354 Freising, Germany; elisabeth.kurze@tum.de (E.K.); vanessa.kock@tum.de (V.K.); 2 Consiglio per la ricerca in agricoltura e l’analisi dell’economia agraria, Unità di ricerca per i processi dell’industria agroalimentare (CREA-IAA), via Venezian 26, 20133 Milan, Italy; roberto.loscalzo@crea.gov.it; 3 Hansabred GmbH & Co. KG, Radeburger Landstr. 12, 01108 Dresden, Germany; k.olbricht@hansabred.org; 4 Humboldt-Universität zu Berlin, Albrecht Daniel Thaer-Institute, 10099 Berlin, Germany

**Keywords:** strawberry, food allergy, Fra a 1, pathogenesis-related proteins, indirect competitive ELISA

## Abstract

Birch pollen allergic patients show cross-reactivity to vegetables and fruits, including strawberries (*Fragaria* × *ananassa*). The objective of this study was to quantify the level of the Fra a 1 protein, a Bet v 1-homologous protein in strawberry fruits by a newly developed ELISA, and determine the effect of genotype, cultivation and food processing on the allergen amount. An indirect competitive ELISA using a specific polyclonal anti-Fra a 1.02 antibody was established and revealed high variability in Fra a 1 levels within 20 different genotypes ranging from 0.67 to 3.97 µg/g fresh weight. Mature fruits of red-, white- and yellow-fruited strawberry cultivars showed similar Fra a 1 concentrations. Compared to fresh strawberries, oven and solar-dried fruits contained slightly lower levels due to thermal treatment during processing. SDS-PAGE and Western blot analysis demonstrated degradation of recombinant Fra a 1.02 after prolonged (>10 min) thermal treatment at 99 °C. In conclusion, the genotype strongly determined the Fra a 1 quantity in strawberries and the color of the mature fruits does not relate to the amount of the PR10-protein. Cultivation conditions (organic and conventional farming) do not affect the Fra a 1 level, and seasonal effects were minor.

## 1. Introduction

Berries are consumed worldwide as fresh fruits as well as processed food products and are an important source of essential nutrients and health beneficial phytochemicals [1]. Strawberry fruits (*Fragaria* × *ananassa, F. × ananassa*) belonging to the group of *Rosaceae* contain high content of vitamin C, folate and phenolic compounds such as anthocyanins, flavonols, flavanols and ellagitannins [2]. Besides their health-beneficial effects e.g., lowering the risk of cardiovascular diseases [3] and cancer [4], strawberry fruits contain proteins, which can elicit food allergies. During the last decades, the prevalence of allergies increased drastically whereby 3–4% of the adult population and up to 5% of children are affected by food allergies [5]. Sensitizations patterns of patients suffering from allergies differ due to diverse geographical distribution of pollen allergens and alimentary habits [6]. In Northern Europe, sensitization against Bet v 1, the major allergen from birch pollen (*Betula verrucosa*), dominates [7]. IgE antibodies produced in response to a primary sensitization to birch pollen allergens can cross-react with similar allergen epitopes from different plant origins leading to birch-pollen related food allergy [8]. Local symptoms affecting the skin (itching) and the mucous membranes (rhinitis) or further more systemic symptoms (asthma, anaphylactic shock) are characteristic afflictions [9]. Food allergies leading to subsequent exclusion of specific fruits and vegetables from a balanced and healthy diet are further associated with a reduced life quality.

Pathogenesis-related (PR) proteins presenting a major source of plant-derived food allergens can be subdivided into 17 different protein families and are widely distributed in the plant kingdom [8]. As part of the plant defense system, expression of *PR-10* genes is induced upon various abiotic and biotic factors such as pathogen attack, wounding, environmental changes and chemicals. PR-10 proteins, including Bet v 1 and homologous allergens from *Rosaceae* such as Mal d 1, the major allergen from apple (*Malus domestica*) or Pru p 1, an allergen from peach (*Prunus persica*), are intracellular proteins with a molecular weight of 16–18 kDa and sensitive to heat and proteases [10]. The widespread occurrence and the high amino acid sequence homology within the PR-10 protein family emphasizes an important function [11].

The genus *Fragaria* comprises numerous species with variations in fruit size and color including red, white and yellow fruits, content of aromatic compounds and genetic diversity with ploidy levels ranging from diploid to decaploid [12]. The octoploid (2*n* = 8*x* = 56) garden strawberry *F*. × *ananassa* Duch. is the primary hybrid-species used for cultivation and commerce and is grown all over the world. The wild members of *F*. *vesca* (2*n* = 2*x* = 14), also known as woodland strawberry, bear small aromatic fruits and are distributed throughout Europe, Northern Asia and North America [13]. *F*. *nilgerrensis* (2*n* = 2*x* = 14), naturally bearing small white fruits, has a fruity banana-like or apricot/peach like aroma [14]. Other species are *F*. × *vescana* (2*n* = 10*x* = 70) obtained by cross-breeding *F*. × *ananassa* × *F*. *vesca*, the Chilean strawberry *F*. *chiloensis* (2*n* = 8*x* = 56) and the musk strawberry *F*. *moschata* (2*n* = 6*x* = 42).

Strawberry fruit are a source of allergens. Currently seven proteins including different isoforms have been found in strawberry fruits (www.allergen.org). They may be responsible for allergenic reactions in sensitized individuals as well as reactions caused due to cross-reactivity of allergens. The non-specific lipid transfer protein Fra a 3 and the profilin Fra a 4 are responsible for strawberry allergies mainly occurring in the Mediterranean area [15], while reactions to the Bet v 1-homologous protein Fra a 1 appear in North and Central Europe [16]. About 30% of patients sensitized to Bet v 1 report allergenic reactions after consumption of strawberry fruits. Compared to fresh fruits, processed food and food products are often tolerated due to heat and protease instability of PR-10 proteins. Bet v 1.0101 (UniProt P15494) and Fra a 1 (UniProt Q3T923) share 53.8% amino acid identity. Fra a 1 shares even 76.3% identity with Mal d 1 (UniProt P43211), the major apple allergen. The three dimensional structure of Fra a 1 shows an identical protein fold with other members of the PR-10 family forming a seven-stranded antiparallel *β*-sheet with two short V-shaped α-helices and a long α-helix at the *C*-terminal [17]. Besides the highly similar glycine-rich loop, the protein forms a hydrophobic cavity indicating a potential role in binding nonpolar molecules. Fra a proteins are able to bind natural flavonoids such as quercetin-3-O-glucuronide and (+) catechin in this hydrophobic cavity [18]. Moreover, the Fra a 1 expression is linked to flavonoid pathway indicating an essential biological role in pigment formation in fruits [19]. In addition, qPCR analysis showed the ripening-related gene expression pattern of *fra a 1.02* and basophil activation tests demonstrated that Fra a 1.02 and Fra a 1.03 proteins resulted in the highest activation of basophils, compared to the other isoforms [16].

As previous studies showed that the allergenic potential of tomatoes [6] and apples [20,21] is cultivar-dependent; the aim of this study was to establish an enzyme-linked immunosorbent assay (ELISA) method for the quantification of the Fra a 1 allergen in strawberry fruits of different genotypes. The influences of genetic effect, cultivation (organic vs. conventional) as well as various processing methods (drying with oven, sun or freeze-drying) on allergen content were be investigated.

## 2. Materials and Methods

### 2.1. Chemicals

All chemicals were purchased from Sigma-Aldrich (Taufkirchen, Germany), Merck (Darmstadt, Germany) and Carl Roth (Karlsruhe, Germany) unless otherwise noted.

### 2.2. Plant Material

In May and June 2017, 20 strawberry cultivars from six species (Table S1) differing in color, size and shape were harvested at full maturity. Plants were grown on the field of Hansabred GmbH & Co. KG (Dresden, Germany) under same light, temperature and water conditions. Fruits were picked at full maturity (day post anthesis 35–40), at the time of the main harvest and stored at −80 °C until analysis.

In the years 2015–2017, *F.* × *ananassa* cultivar Asia, a well-known cultivar used in organic cultivation, was grown either conventionally or organically in Forlì-Cesena, Italy (lat. 44°13′15.91″ N; long. 12°14′41.27″ E). The specific details for growing conditions are listed in Tables S2 and S3. Fruits were harvested at full maturity (35–40 days post anthesis) and divided in four aliquots, one was directly stored at −80 °C (fresh) and the remaining three were subjected to different drying processes (freeze, oven and sun).

### 2.3. Heterologous Expression and Purification of rFra a 1.02 Protein

The plasmid construct pQE70 *fra a 1.02* generated in a previous study [16,22] was used for transformation in the expression strain *Escherichia coli* (*E*. *coli*) BL21 (DE3) pLysS. Recombinant Fra a 1.02 was expressed as a fusion protein with a *C*-terminal His-tag. Cells were grown in 1 l LB medium containing 100 µg/mL ampicillin and 34 µg/mL chloramphenicol at 37 °C until optical density reached 0.6. After induction of gene expression with 1 mM isopropyl *β*-d-1-thiogalactopyranoside, (IPTG) the culture was further incubated for 20 h at 18 °C. Cells were harvested by centrifugation (10 min; 5282 g; 4 °C) and cell pellets were stored at −80 °C. The purification of the recombinant protein was performed via immobilized metal affinity chromatography (IMAC) using the Profinity IMAC Resin (Biorad). After suspending the pellet in 10 mL binding buffer (30 mM Tris-HCl pH 7.5; 500 mM NaCl; 15 mM imidazole) supplemented with 1 mM phenylmethylsulfonyl fluoride (PMSF) and *β*-mercaptoethanol, ultrasonification and centrifugation (30 min; 21,191 g; 4 °C), the supernatant containing soluble proteins was incubated for 2 h at 4 °C with the IMAC Resin. Following two washing steps with 10 mL of binding buffer, rFra a 1.02 protein was finally eluted with elution buffer (30 mM Tris-HCl pH 7.5; 500 mM NaCl; 250 mM imidazole). The purity of the protein fractions was evaluated by SDS-PAGE. Five µg protein was separated in a 12% acrylamide stacking gel at 100 V for 2.5 h under reducing conditions with *β*-mercaptoethanol. Protein staining was performed with Coomassie Brilliant Blue G250. As molecular weight marker PageRuler Prestained Protein Ladder (Thermo Scientific, Planegg, Germany) was used. Pure elution fractions were pooled and dialyzed against carbonate buffer (pH 9) at 4 °C for 20 h. Insoluble particles were removed by centrifugation. After freeze-drying, proteins were dissolved in Milli Q, containing 1 mM l-cysteine and stored at −20 °C and were further used as a standard substance for indirect competitive ELISA.

In addition, refolding of the rFra a 1.02 protein after denaturation with urea and antibody binding was examined by purification of the protein from the insoluble fractions (inclusion bodies). Therefore, the remaining cell pellet after cell lysis, ultra-sonication and centrifugation was dissolved in denaturation buffer (30 mM Tris-HCl pH 7.5; 0.5 M sodium chloride; 15 mM imidazole; 8 M urea) at 4 °C over-night. After centrifugation (30 min; 21,191 g; 4 °C) proteins were refolded via dialysis against refolding buffer (30 mM Tris-HCl pH 7.5; 0.5 M sodium chloride; 15 mM imidazole), at 4 °C over-night and centrifuged again to remove insoluble proteins. The supernatant was incubated with IMAC resin for 2 h at 4 °C and protein purification was performed as described for the soluble protein fraction. The purity of the protein fractions was examined by SDS-PAGE followed by Coomassie staining and IgG binding capacity was analyzed by Western blot using 2 µg/mL of a primary polyclonal antibody with specificity against Fra a 1.02 and a secondary Anti-Rabbit-Alkaline Phosphatase (AP) antibody diluted 1:7500 (*v*/*v*).

### 2.4. Protein Determination

The total protein concentration was quantified using Roti^®^-Nanoquant following the manufacturer’s instructions (Carl Roth, Germany). Measurement was performed in microtiter plates (Greiner 96 well plates, polypropylene, Sigma-Aldrich, Taufkirchen, Germany). Bovine serum albumin (BSA) was used as standard protein. Absorption at 450 nm and 590 nm was measured with the CLARIOstar plate reader (BMG Labtech, Germany).

### 2.5. Production of Polyclonal Anti-Fra a 1.02 Antibody

Davids Biotechnologie GmbH (Regensburg, Germany) produced the polyclonal antibody with specificity against Fra a 1.02. Elution fractions of purified rFra a 1.02 protein purified from soluble protein fraction were used for immunization of rabbits comprising six immunizations within 63 days. Furthermore, the antiserum was purified via affinity chromatography using a Fra a 1.02 bound column. Finally, the purified polyclonal antibody (0.9 mg/mL) was used for Western blot analysis and ELISA. As secondary antibodies, Anti-Rabbit-Horseradish peroxidase (HRP) and Anti-Rabbit-Alkaline Phosphatase (AP) were purchased from Carl Roth (Germany).

### 2.6. Thermal Treatment of rFra a 1.02

Freeze-dried rFra a 1.02 from soluble and insoluble fraction was diluted in PBS (pH 7.4) to five µg total protein and further incubated for 10, 30, 60 and 90 min at 99 °C in a Thermoblock (Thermomixer comfort, Eppendorf), and cooled on ice immediately. As control, non-heated protein solution was used. The integrity of the protein was analyzed by SDS-PAGE and IgG binding was examined via Western blot using the specific polyclonal anti-Fra a 1.02 antibody.

### 2.7. Processing of Strawberry Fruits

Strawberries from cultivar Asia (*F*. × *ananassa* Duch.) were harvested at full maturity in May 2015 to 2017 and were further subjected to drying. Ripe fruits were cut into halves and dried using three different methods upon constant dry weight (DW). Oven and solar-dried fruits were only available in 2015 and 2016. For oven drying a conventional oven dryer (Thermo-Lab, Codogno, Italy) was used. Drying process was performed at 55 °C for 72 h. Solar drying via solar irradiance was performed in a miniaturized plant (TermoTend System-GTek, Carpi, Italy) for 7 to 10 days. Temperature varied between 22 °C and 35 °C due to day-night-cycle. Freeze-drying was performed from −35 °C to room temperature for 96 h using an air-forced tunnel and a Dura-Stop tray dryer combined with a Dura-Dry condenser module for lyophilization (FTS Systems, Stone Ridge, New York, NY, USA). Freeze-dried samples were further powdered before storage. Water loss was calculated from the weights of fresh and dried samples. All samples were stored at −20 °C until analysis.

### 2.8. Strawberry Extracts

Proteins from fresh and dried strawberry fruits were extracted according to Vassilopoulou [23]. To reduce the allergen variability within one fruit and between fruits, eight frozen strawberry fruits of one genotype were cut into small pieces, pooled and grind to a fine powder using a commercial blender (Personal Blender PB 250). For each sample protein extraction was performed in triplicates. Strawberry powder was homogenized with extraction buffer (200 mM potassium phosphate buffer pH 7; 20 mM ethylenediamine tetraacetic acid (EDTA); 100 mM diethyldithiocarbamic acid (DIECA); 5% (*w*/*v*) polyvinylpolypyrrolindone (PVPP); 0.5% (*v*/*v*) Tween 20) containing 0.5 mM PMSF and protease inhibitor cocktail (Complete Protease Inhibitor Cocktail, Roche). For fresh fruits, a ratio of 1:2 (*w*/*v*) was applied, whereas for dried strawberries a ratio of 1:8 (*w*/*v*) was used to warrant proper mixing. After incubation for 30 min at 4 °C and end-over-end rotation, insoluble particles were removed by centrifugation (15 min; 5292 g; 4 °C). The supernatant containing soluble proteins was dialyzed against phosphate-buffered saline (PBS) (pH 7.4) over night at 4 °C (ZelluTrans 3.5 kDa MWCO, Carl Roth). To remove other insoluble precipitates, a second centrifugation (10 min; 16,100 g; 4 °C) was performed and extracts were directly used for allergen determination via indirect competitive ELISA.

### 2.9. Temperature Stability of Protein Extracts

Protein extracts from *F.* × *ananassa* cultivars Elsanta and Snow White, *F.* × *vescana* cultivar Florika and *F. vesca* cultivar Yellow Wonder were incubated for 10 min at 30, 40, 50, 60, 70, 80, and 90 °C in a Thermoblock (Thermomixer comfort, Eppendorf, Germany), and cooled on ice. As control, non-heated protein extract was used. The stability of the native Fra a 1 protein was further analyzed by indirect competitive ELISA.

### 2.10. Indirect Competitive ELISA

The Fra a 1 content of strawberry samples was quantified by indirect competitive ELISA using rFra a 1.02 as standard protein and competitor and a specific polyclonal anti-Fra a 1.02 antibody. Microtitre plates (immunoGradeTM Brand) were coated with 100 µL of 0.5 µg/mL rFra a 1.02 diluted in coating buffer (PBS pH 7.4) and incubated at 4 °C over-night. After three washing steps with 300 µL of washing buffer (0.05% (*v*/*v*) Tween 20 in PBS), free binding sites were blocked with 200 µL of 1% milk powder diluted in PBS for 2 h at room temperature and washed as before. For each strawberry sample protein extraction was performed in triplicates and analyzed on the same microtitre plate. Fifty µL of dialyzed strawberry protein extracts were diluted (1:2 and 1:4) in washing buffer and pipetted in triplicates to each well as “free” Fra a 1. For competition between free and immobilized allergen, 50 µL of 1 µg/mL polyclonal anti-Fra a 1.02 antibody solution, diluted in washing buffer, was added and incubated for 3 h at 4 °C. To remove unbound primary antibody, plates were washed four times and sequentially incubated with 100 µL of 1 µg/mL Anti-Rabbit-HRP (Carl Roth, Germany) for 1 h at room temperature. After a final washing step, 100 µL 1-Step Ultra 3,3′,5,5′-tetramethylbenzidine (TMB) ELISA solution was added and color development was performed for 10 min in the dark. The enzymatic reaction was stopped with 100 µL 2 M sulfuric acid and absorption was detected at 450 nm and 620 nm using the CLARIOstar plate reader (BMG Labtech, Germany). Blank well controls and standard curves were applied on each microplate. The standard curve was measured with serial dilutions of 0.0001–50 µg rFra a 1.02/mL. Fifty µL/well of rFra a 1.02 was pipetted as “free” allergen and followed by the same procedure as the strawberry samples. Data analysis was performed using the MARS software (BMG Labtech, Germany). Allergen amount in strawberry extracts was quantified with 4-Parameter Plot and expressed as µg Fra a 1/g FW respectively µg Fra a 1/g DW. Dry matter was converted to fresh weight (FW) considering the loss of water in percent during drying process.

### 2.11. Statistical Analysis

The statistical analysis software R (The R Foundation for Statistical Computing, R version i386 3.5.0) was used for data analysis and illustration of box plots. Experimental data were analyzed using the nonparametric Kruskal–Wallis test. Median values and statistical significance levels between the variable groups were calculated using Dunn’s Test and p-value adjustment according to Bonferroni method. *p* values of ≤0.05 were considered as significant.

## 3. Results

### 3.1. Purification and Thermal Stability of Recombinant Fra a 1.02 Protein from Soluble and Insoluble Fraction

**Figure 1:**
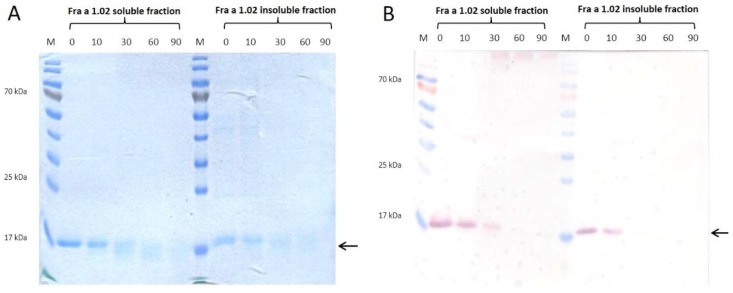
Heat stability of recombinant Fra a 1.02. (**A**) SDS-PAGE performed under reducing conditions with *β*-mercaptoethanol and (**B**) Western blot analysis of dialyzed elution fractions of the rFra a 1.02 protein purified from soluble and refolded insoluble fraction. Two µg of protein were heated for 10, 30, 60 and 90 min at 99 °C. Untreated protein served as control (0). Coomassie Brilliant Blue G250 was used for protein staining. For Western blot specific polyclonal anti-Fra a 1.02 antibody was used. M: PageRuler Prestained Protein Ladder.

Recently, eight isoforms of the Fra a 1 protein have been identified as Bet v 1-related allergens in *F.* × *ananassa* [16]. The gene encoding the isoform Fra a 1.02 is highly expressed in the fruit tissue compared to the low expression level of Fra a 1.01E and 1.03; Fra a 1.01E and Fra a 1.03 are highly expressed in roots and open flowers, respectively. In general, their relative expression levels are low [19]. Besides the high relative expression and the highest activation of basophils among all eight isoforms sharing 74.5–97.5% amino acid sequence identity [16], Fra a 1.02 was identified as the major strawberry allergen and was therefore selected for the purpose of this study. Visualized by SDS-PAGE, the rFra a 1.02 produced in *E. coli* BL21 (DE3) pLysS as a *C*-terminal His-tag fusion protein showed one dominant band at the predicted molecular weight of 18 kDa in the eluted fractions of both, soluble and refolded insoluble fraction (Figure 1A). The polyclonal anti-Fra a 1.02 antibody was produced upon immunization of rabbit with the purified recombinant protein from the soluble fraction. IgG antibodies recognized the soluble as well as the refolded rFra a 1.02 form as confirmed by Western blot (Figure 1B). In addition, different isoforms of the native Fra a 1 contained in protein extracts isolated from fruits of different strawberry varieties were specifically recognized among total soluble protein (Figure S1).

To investigate the effect of heat on IgG recognition, dialyzed elution fractions of rFra a 1.02 purified from soluble and refolded insoluble protein fractions were thermally treated and examined by SDS-PAGE (Figure 1A) and Western blot (Figure 1B). After 10 min at 99 °C rFra a 1.02 protein was still detectable showing a band at 18 kDa in the Coomassie stained gel (Figure 1A). After a prolonged heating process for 30, 60 and 90 min the protein band became more and more blurred. In accordance with the SDS-PAGE, IgG-binding activity decreased after 30 min of thermal treatment showing barely visible protein bands (Figure 1B). Consequently, after 30 min at 99 °C no bands were visible.

### 3.2. Establishment and Validation of the ELISA

Using the rFra a 1.02 as a solid phase-bound antigen and as a standard protein, an indirect competitive ELISA was established to determine the Fra a 1 content in strawberries. A specific polyclonal Fra a 1.02-rabbit antibody was used to quantify the Bet v 1-homologous in strawberry extracts. The standard curve of the ELISA evaluated with the MARS software showed a typical linear range from 0.01 to 10.0 µg/mL with reproducible results (Figure S2). The inter-assay variation of the absorption values of the standard curve (serial dilutions of 0.0001–50 µg rFra a 1.02/mL for competition reaction) performed in triplicates were under 5%. The intra-assay (day-to-day/plate to plate) variation was under 20%. Standard curves were similar and, therefore, resulting data of strawberry samples with unknown Fra a 1 content could be compared. The established ELISA method remained unaffected by small variations of parameters (room temperature, preparation of buffer solution). Indicated by the decrease of the absorption signal of the four-parameter plot, the lowest concentration of the target protein Fra a 1 was 0.01 µg/mL reflecting the detection limit. Strawberry extracts were diluted to a concentration within the working range and gave reliable results demonstrating the dilutional linearity.

### 3.3. Influence of Strawberry Variety on Fra a 1 Content

**Figure 2:**
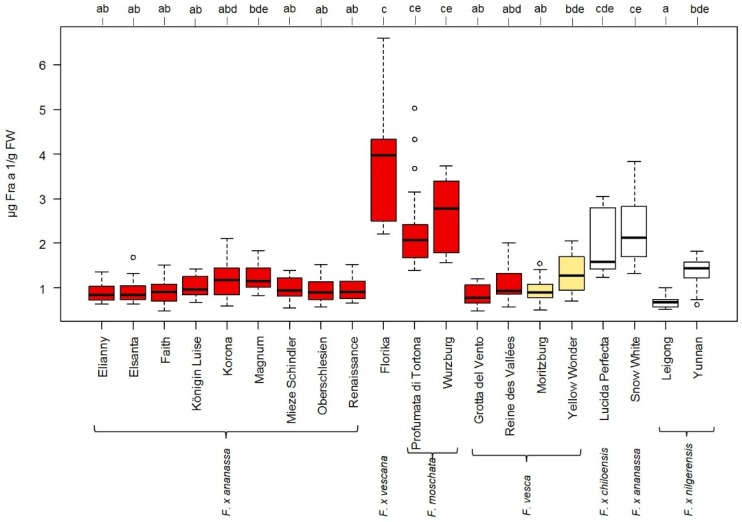
Fra a 1 content in fruits of different strawberry genotypes. Allergen content in µg Fra a 1/g FW determined with indirect competitive ELISA. The color of the box plots corresponds to the color of the ripe strawberry fruits. Comparisons of median values were performed by the Dunn’s Test. Significant differences for each cultivar were calculated at a significance level of 5% (*p* value ≤ 0.05) indicated with letters a–d.

Fruits of twenty different *Fragaria* genotypes including mature red-, white-, and yellow-colored fruits of different sizes were investigated regarding the genetic effect (cultivar-to-cultivar) on Fra a 1 expression at the translational level (Figure 2). An established method was used for protein extraction [23]. The total soluble protein level of fresh strawberry fruits varied between 0.89 and 2.04 mg soluble protein/g FW (Table S1). The Fra a 1 concentration ranged from 0.67 to 3.97 µg/g FW (median value) comprising five significance groups (letters a–e) with 5% of significance level (*p* value ≤ 0.05) according to the Dunn’s Test (Figure 2). The *F.* × *vescana* cultivar Florika showed the highest level of Fra a 1 (3.97 µg/g FW) followed by *F. moschata* accession Wuerzburg with 2.78 µg/g FW. The lowest concentration was determined in *F. nilgerrensis* accession Leigong and *F. vesca* accession Grotta del Vento with 0.67 and 0.78 µg/g FW, respectively. Leigong showed a 5.9-fold lower Fra a 1 content compared to Florika. The *F.* × *ananassa* genotypes contained a similar level of the allergen ranging between 0.84 and 1.17 µg/g except for the white-fruited *F.* × *ananassa* cultivars Snow White with significant higher Fra a 1 content of 2.13 µg/g FW and Lucida Perfecta with 1.58 µg/g FW. Even the red-fruited genotypes of *F. vesca* Grotta del Vento and Reine des Vallées as well as the yellow-fruited *F. vesca* Moritzburg revealed allergen levels similar to those of *F.* × *ananassa* genotypes. *F. vesca* Yellow Wonder, *F. chiloensis* Lucida Perfecta and *F. nilgerrensis* Yunnan showed slightly higher Fra a 1 levels than the *F.* × *ananassa* cultivars. The percentage of Fra a 1 referred to the total soluble protein amount ranged from 0.04% for Leigong to 0.28% for Florika (Table S1).

### 3.4. Effect of Cultivation and Processing Methods on Allergen Content in Strawberry Fruits

**Figure 3:**
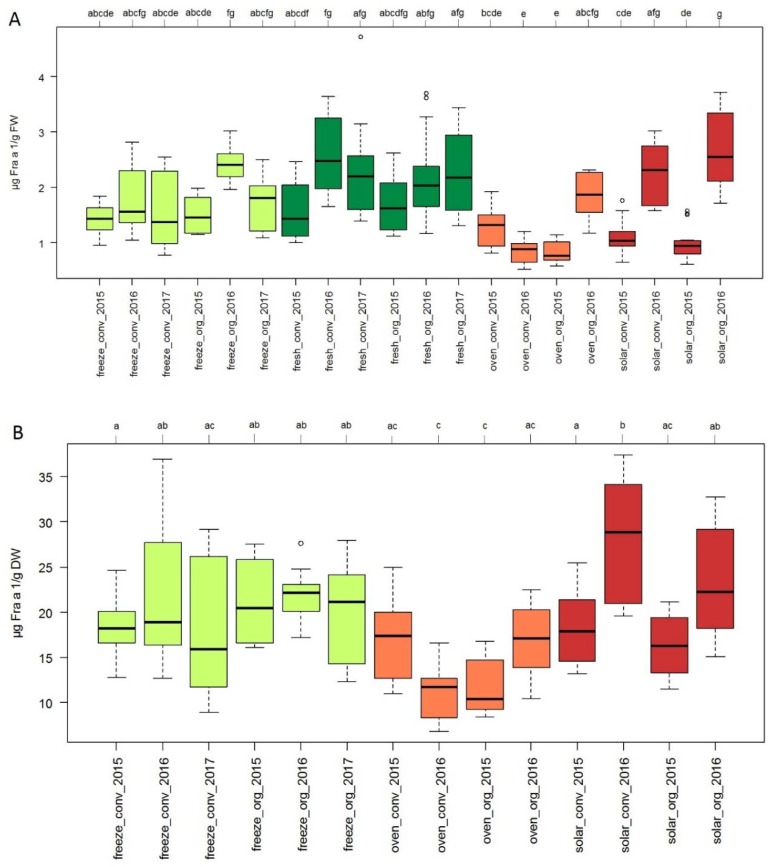
Fra a 1 content in fresh and dried fruits of strawberry cultivar Asia. (**A**) Allergen content in µg Fra a 1/g FW and (**B**) µg Fra a 1/g DW determined with indirect competitive ELISA. Plants were grown in Italy in three consecutive years from 2015 to 2017 either conventional (conv) or organic (org). Ripe fruits (fresh) were dried via freeze-drying (freeze), in the oven (oven) and in the sun (solar). Comparisons of median values were performed by the Dunn’s Test. Significant differences for each group were calculated at a significance level of 5% (*p* value ≤ 0.05) indicated with different letters (a–g).

The effects of cultivation conditions, seasonal effects and processing techniques such as drying on the Fra a 1 content were examined for *F.* × *ananassa* cultivar Asia (Figure 3A). Cultivar Asia was chosen as it is tolerant against most common root-diseases and thus, used in organic farming.

Statistical evaluation of the data yielded seven significance groups (letters a–g) with 5% significance level (*p* value ≤ 0.05) according to the Dunn’s Test when the Fra a 1 content of the samples was expressed as µg/g FW. The Fra a 1 level ranged from 0.77 µg/g FW for oven-dried fruits grown organically in 2015 up to 2.54 µg/g FW for solar-dried samples grown organically in 2016. In freeze-dried samples Fra a 1 levels between 1.38 and 2.4 µg/g FW were detected, which were similar to the concentrations found in fresh fruits ranging from 1.44 and 2.48 µg/g FW. Two out of eight oven and solar-dried samples showed significantly lower Fra a 1 concentrations compared to fresh fruits.

Three significance groups (letters a–c) were obtained when the Fra a 1 values were expresses as µg/g DW. In dried samples the Fra a 1 content varied between 10.4 and 28.8 µg/g DW for oven-dried fruits grown organically in 2015 and solar-dried fruits grown conventionally in 2016 (Figure 3B). Freeze-dried samples showed no significant differences with similar Fra a 1 content ranging from 16 to 22.1 µg/g DW in all three years. Furthermore, no significant differences between cultivation techniques, either organic or conventional, were found for all dried samples. Both, seasonal effects and effects of cultivation conditions were observed for oven dried samples. Moreover, solar-dried strawberries showed year-dependent Fra a 1 levels. Although the growing method had no influence on solar-dried fruits, seasonal variations had an impact, whereby the allergen content in 2016 was significantly higher than in 2015.

### 3.5. Temperature Stability of Native Fra a 1 in Strawberry Protein Extracts

The temperature stability of Fra a 1 in protein extracts isolated from *F.* × *ananassa* cultivars Elsanta and Snow White, *F.* × *vescana* cultivar Florika and *F. vesca* cultivar Yellow Wonder was investigated.

**Figure 4:**
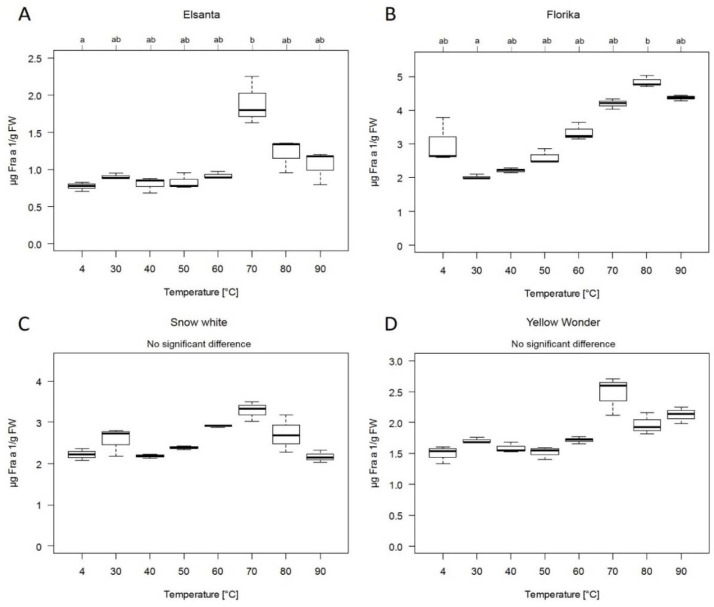
Thermal stability of native Fra a 1. Allergen content of strawberry cultivar (**A**) Elsanta, (**B**) Florika, (**C**) Snow White and (**D**) Yellow Wonder in µg Fra a 1/g FW. Strawberry extracts were heated for 10 min at increasing temperatures from 30 to 90 °C. Untreated extract served as control (4 °C). Fra a 1 content was determined with indirect competitive ELISA. Comparisons of median values were performed by the Dunn’s Test. Significant differences for each group were calculated at a significance level of 5% (*p* value ≤ 0.05) indicated with letters a and b.

Extracts were heated for 10 min at different temperatures ranging from 30 to 90 °C followed by allergen quantification via ELISA (Figure 4). Up to 50 °C Fra a 1 level of the different samples was rather constant, except for Florika. The detected Fra a 1 content increased for all cultivars at higher temperatures (70 °C) compared to the untreated 4 °C control. The highest Fra a 1 level was detected at 70 °C for cultivars Elsanta, Snow White and Yellow Wonder (Figure 4A,C,D) and at 80 °C for cultivar Florika (Figure 4B).

## 4. Discussion

Food allergies especially to *Rosacea* fruits such as apple, peach and strawberry are often associated with birch pollen-related allergies due to cross-reacting IgE antibodies [24]. About 70% of birch-pollen allergic patients show IgE-mediated food allergies against various fruits and vegetables [25]. Strawberry allergy mainly affects 30% of population revealing adverse reactions after ingestion of the fruit [26]. The Bet v 1-homologous Fra a 1 is recognized by IgE from strawberry allergic patients [27] and cross-reacts with antibodies to Bet v 1 and the homologous apple allergen Mal d 1 (61% and 78% sequence identity, respectively). Here, we report the first ELISA applicable for the quantification of Fra a 1, a member of the PR-10 family in strawberry fruit. Studies showed that Fra a 1 constitutes a protein family of at least eight members according to the corresponding sequences found in the *F. vesca* and *F.* × *ananassa* genomes [16]. Since isoform Fra a 1.02 showed high basophil activation in BAT and its coding gene is highly expressed in ripe fruits, Fra a 1.02 was identified as important allergen in strawberry fruits and selected as target protein. Due to the use of the polyclonal antibody raised against Fra a 1.02, it is to be assumed that other isoforms of the same protein in strawberry fruit extracts can be recognized. Therefore, the determination of the total Fra a 1 amount is possible.

### 4.1. Biochemical Properties and Thermal Stability of Recombinant Fra a 1.02

SDS-PAGE analysis of the rFra a 1.02 protein under reducing conditions showed a distinct band with a molecular weight of 18 kDa (Figure 1). The polyclonal anti-Fra a 1.02 antibody showed binding capacity to the protein purified from the soluble fraction as well as refolded from the insoluble fraction (Figure 1). Thus, denaturation with urea and refolding via dialysis allowed recognition of essential epitopes for antigen-antibody binding (Figure 1). Formation of inclusion bodies of recombinant proteins is a frequently observed phenomenon as overextension of the protein translation and folding machinery in *E. coli* may lead to misfolded, inactive protein aggregates whereby the content of soluble proteins decreases. A solution is the purification of the inclusion bodies under denaturing conditions and refolding of the target protein. This procedure delivers the protein in high yield and purity. Furthermore, depending on the conditions during bacterial cultivation, correctly folded secondary structure elements can be present [28]. Recombinant proteins isolated from inclusion bodies and natural allergens may show similar allergenic activities, as demonstrated for Pyr c 1, the Bet v 1-homolog from pear [29]. Specific IgE sera from patients allergic to pear recognized the recombinant Pyr c 1 refolded from insoluble fraction. In addition to soluble and refolded rFra a 1.02 the polyclonal anti-Fra a 1.02 antibody also specifically recognized native Fra a 1 from strawberry extracts as verified by Western blot (Figure S1). Except for an unknown cultivar, all other protein extracts from fruits of known strawberry cultivars showed a single visible band in Western blot whereas native Fra a 1 was barely visible by Coomassie staining.

The rFra a 1.02 protein is thermally unstable (Figure 1). Due to heat treatment at 99 °C, simulating the cooking process, the protein structure of the recombinant protein changed, so that recognition and binding of the specific polyclonal antibody was remarkably reduced or even not possible (after 60 and 90 min) depending on the time of heat treatment. Likewise, other PR-10 proteins, such as rPru av 1 from cherry [30] and Mal d 1 from apple [31] are heat sensitive. A common feature of PR-10 proteins is the thermal instability and enzymatic digestion by proteases within the lower part of the gastrointestinal tract [30]. After ingestion of fruits from *Rosaceae* family, symptoms of allergic reactions to PR-10 proteins are restricted to the upper part of the intestinal tract (mouth). Remarkably, the native strawberry Fra a 1 seems to be more heat stable in the strawberry protein extract after short-term heating for 10 min up to 90 °C (Figure 4). Both, recombinant Fra a 1.02 and fruit extracts were diluted or dialyzed in PBS (pH 7.4), respectively. The matrix composition of the extract or binding of natural ligands may have a stabilization effects underlining the function of Fra a 1 proteins in pigment formation and binding of compounds from the secondary metabolic pathway such as quercetin-3-O-glucuronide or (+) catechin [18,19]. Similarly, heating of semi-purified protein extracts from celery tuber and apple for 30 min at 100 °C did not deplete the immune-reactivity of the major allergens in these samples [31]. In our study, in some cases, the results of the indirect competitive ELISA demonstrated an even higher Fra a 1 content after heating (10 min at 70 °C). The detected increase of Fra a 1 might be due to changes in protein structure after thermal treatment leading to the presentation of essential epitopes for recognition by the specific antibody. Thus, additional Fra a 1 isoforms, besides Fra a 1.02, which might be present in the strawberry protein extracts, could present extra epitopes due to heat-induced conformational changes [32,33]. Nevertheless, prolonged heating resulted in complete protein destruction [30] as shown for rFra a 1.02 in buffer solution (Figure 1). Although Bet v 1 homologous proteins have been generally reported to be sensitive to heat the overall thermal instability might vary due to the primary and secondary structure but also due to the environment in which the protein is heated, e.g., sugar rich solution.

### 4.2. Fra a 1 Content of Strawberry Fruits Is Genotype Dependent

The variety within the genus *Fragaria* is enormous with diversity in color, size, aroma compound composition and ploidy levels [12]. The fruit color has been correlated with allergen content [34]. Although colorless strawberry cultivars have low commercial importance due to some negative features (small and soft fruits), they attracted attention as two white-fruited cultivars showed low Fra a 1 levels in comparison to red-fruited varieties when examined by 2D-DIGE [35]. Hence, the newly developed ELISA was used to quantify the Fra a 1 content in various strawberry genotypes. Among 20 varieties differing in genus, color and size, the Fra a 1 level varied significantly confirming the genotype dependency (Figure 2). Previous studies obtained similar results and proved that the allergenicity of apples and tomatoes is cultivar dependent [20,36]. All red-fruited cultivars from *F.* × *ananassa* revealed a similar Fra a 1 content, except for the white-fruited cultivar Snow White, which showed a significant higher Fra a 1 concentration (Figure 2). Snow White was developed by cross-breeding with Chilean landraces of *F. chiloensis*. This pedigree background might be one reason for the high Fra a 1 content, since the *F. chiloensis* cultivar Lucida Perfecta also showed levels higher than that of the red-fruited *F.* × ananassa. Of all investigated cultivars, the highest Fra a 1 level was determined in Florika *F.* × *vescana* obtained by cross-breeding *F.* × *ananassa* × *F. vesca*. Due to crossing of cultivated strawberries with wild species nutritional properties may altered as well as unfavorable compounds such as food allergen [37]. Overall, the colorless fruited cultivars showed varying Fra a 1 levels between 0.67 and 2.13 µg/g FW verifying recent results, that white-fruited strawberries do not always show reduced Fra a 1 levels [12]. Despite of the white fruit color of Lucida Perfecta, a representative of *F. chiloensis*, a relative high allergen content of 1.58 µg Fra a 1/g FW was quantified. Plants of the species *F. chiloensis* have been considered resistant against frost and diseases by some breeders and were therefore used as parents for crossing [13]. Robustness and resistance of plants is often related to the expression of PR-10 protein because plants synthesize them in response to different stress factors.

Basophil activation tests demonstrated that fruits from white-fruited cultivars have similar allergenic potential compared to red-fruited cultivars [12]. Similarly, our results show that the Fra a 1 level in fruits of the cultivated strawberry genotype *F.* × *ananassa* is not statistically significant to the concentration in yellow- and white-fruited genotypes, except Lucida Perfecta and Snow White, which even show higher values. However, it is impossible to conclude on the general allergenicity of the fruits from the different genotypes as allergic reactions are complex processes differing from patient to patient and occurring symptoms cannot be attributed to one single protein. Previous studies showed that the content of the major apple allergen Mal d 1, a Bet v 1- and Fra a 1-homolgue, is not directly related to the allergenic response of birch pollen allergenic patients due to patient-related variations [38]. In addition, various Fra a 1 isoforms are produced in strawberry fruits during maturation and ripening, which show different binding affinities to the polyclonal antibody and differ considerably in basophil activation [16]. For allergenic patients the reaction to a specific PR-10 isoform might be different depending on the IgE-antibodies from patient sera.

### 4.3. Cultivation Condition and Drying Processing of Strawberries has Minor Effect on Fra a 1 Content

Thermal treatment of food like cooking, boiling or dry heating is an essential process in daily life as well as in food industry. Allergenicity of food can be altered by increasing, unchanging or decreasing the allergen amount, depending on the structure and features of allergic proteins [39]. Since dried fruits are a common product in food industry, especially in cereal, the Fra a 1 amount was quantified in various dried strawberries. Allergic reaction in patients cross-reacting to Bet v 1-homologous proteins of the *Rosaceae* family are primarily caused by fresh fruits and symptoms are mainly restricted to the mouth (OAS) due to the sensitivity to heat and proteases. This implies that processed food or food products might be tolerated [40]. For native Mal d 1 [31] and recombinant Pru av 1 [30] a loss of allergenicity due to thermal processing was observed. In this study we showed that rFra a 1.02 is heat-sensitive in buffer solution (Figure 1) whereas native Fra a 1 was quite stable in a strawberry protein extract (Figure 4).

Changes in micronutrient content, phenolic compound composition acting as natural antioxidants as well as allergen level in strawberry fruits might be explained by a genotype-dependent reaction to various environmental stress-conditions differing from year to year [41]. The influence of cultivation conditions, seasonal effects and drying processes on the Fra a 1 allergen content was examined by indirect competitive ELISA. For fresh strawberries from *F.* × *ananassa* Asia, no significant differences were obtained comparing the two growing methods organic vs. conventional of the same year (Figure 3A). Seasonal effects were minor and a drastically reduced level of the Fra a 1 was not observed in dried strawberries. The average temperature for drying in the oven was 55 °C. Depending on the sun intensity, the daily temperature varied between 22 and 35 °C for solar drying. Although the protein structure of the native Fra a 1 protein might be changed due to the loss of water during drying, the antibody is still able to recognize the protein or fragments. The temperature rise during processing seems not sufficient to completely destroy essential epitopes. As shown for rFra a 1.02 only prolonged heating at high temperatures results in complete protein destruction and loss of IgG activity (Figure 1). Similarly, the gentle method of freeze-drying known to preserve the protein structure did not affect the Fra a 1 level compared to fresh fruits (Figure 3A).

The meteorological data (Table S3) from the growing location in Italy at Forlì-Cesena presenting the main ripening seasons in April and May revealed a progressive increase in temperature and a decrease in relative humidity from April to May. Temperatures were generally higher in April 2016 whereas atmospheric moisture was higher in May 2016 compared with the other years. Variations of the temperature and humidity might have affected the sensitive signaling pathways within the fruits and consequently the amount of Fra a 1. Thus, varying weather conditions including changes in temperature, precipitation and humidity as well as pathogen infestation seem to have a more important effect on the Fra a 1 level than growing methods. Specific climatic conditions can promote pathogen growth leading to synthesis of PR-10 proteins. This well-known plant response might eventually explain the observed seasonal effect, which is especially obvious for the solar-dried samples. In 2016, the pathogen pressure might be quite intense due to the high humidity and resulted in the highest Fra a 1 levels quantified in the study.

## 5. Conclusions

The content of the Bet v 1-related Fra a 1 protein in strawberry fruits varied significantly between different genotypes whereby varieties from one species show similar levels. Species showing a reduced Fra a 1 content might be beneficial for breeding of new cultivars with improved fruit quality. The effect of the cultivation method on the Fra a 1 content was insignificant but seasonal variations were observed. The rFra a 1 protein was stable against short-term thermal treatment but was degraded and lost IgG binding activity after prolonged heating.

## Supplementary Materials

The following are available online at http://www.mdpi.com/2072-6643/10/7/857/s1, Figure S1: Protein pattern of strawberry extracts, Figure S2: Standard curve of indirect competitive ELISA, Table S1: Strawberry cultivars, Table S2: Growing conditions for conventional and organic strawberry cv. Asia, Table S3: Temperature and relative humidity at the growing location Forlì-Cesena in Italy for April and May in the years 2015 to 2017.
