# Supplementary material for: Effect of the Strawberry Genotype, Cultivation and Processing on the Fra a 1 Allergen Content
Source: Nutrients. 2018 Jul 2;10(7):857. doi: 10.3390/nu10070857 (PMC6073608; doi:10.3390/nu10070857)
Supplement: Supplementary file 1 [file nutrients-10-00857-s001.pdf]

nutrients

Supplemental data

Effect of the strawberry genotype, cultivation and processing on the Fra a 1 allergen content

Elisabeth Kurze [1], Vanessa Kock [1], Roberto Lo Scalzo [2], Klaus Olbricht [3,4], Wilfried Schwab [1,*]

[1] Biotechnology of Natural Products, Technische Universität München, Liesel-Beckmann-Str.1, 85354 Freising, Germany1; elisabeth.kurze@tum.de; vanessa.kock@tum.de; wilfried.schwab@tum.de

[2] Consiglio per la ricerca in agricoltura e l'analisi dell'economia agraria, Unità di ricerca per i processi dell'industria agroalimentare (CREA-IAA), via Venezian 26, 20133 Milan, Italy; roberto.loscalzo@crea.gov.it

[3] Hansabred GmbH& Co. KG, Radeburger Landstr. 12, 01108 Dresden, Germany; k.olbricht@hansabred.org
[4] Humboldt-Universität zu Berlin, Albrecht Daniel Thaer-Institute, Berlin, Germany

Figure S1. Protein pattern of strawberry extracts.

Figure S2. Standard curve of indirect competitive ELISA.

Table S1. Strawberry cultivars

Table S2. Growing conditions for conventional and organic strawberry cv. Asia cultivated in Italy, Forlì-Cesena district.

Table S3. Temperature and relative humidity at the growing location Forlì-Cesena in Italy for April and May in the years 2015 to 2017; National Agrometereological DataBase of Italy SIAN (National Agricultural Information System)

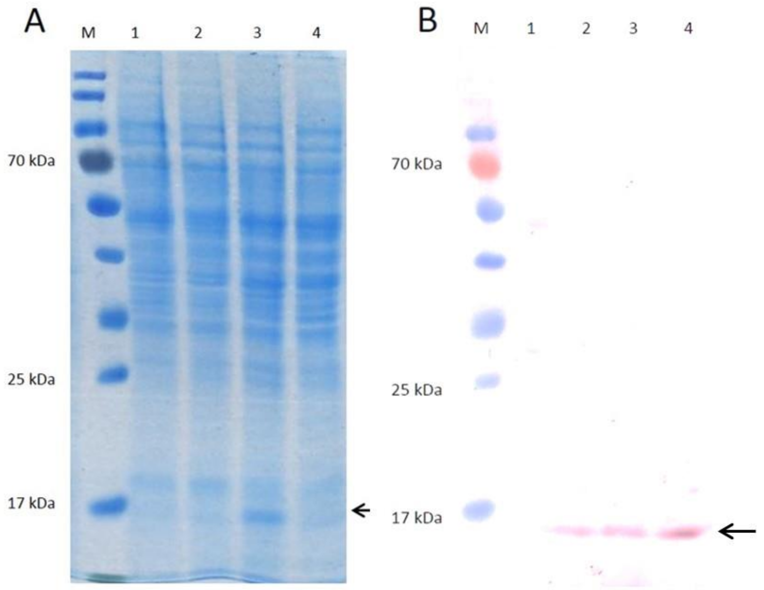

Figure S1. Protein pattern of strawberry extracts.

Native strawberry protein extracts of commercially available cultivars analyzed by (A) SDS-PAGE and (B) Western-Blot analysis. 1 – Fruits (unknown cultivar) from grocery store strawberry, 2 – Elsanta, 3 – Florika, 4 – Magnum. SDS-PAGE was performed under reducing conditions. For protein staining coomassie Brilliant Blue G250 was used. Western blot analysis was performed using a specific polyclonal Fra a 1.02-antibody. The 18 kDa band, corresponding to the native Fra a 1, is marked with an arrow. M: PageRuler Prestained Protein Ladder

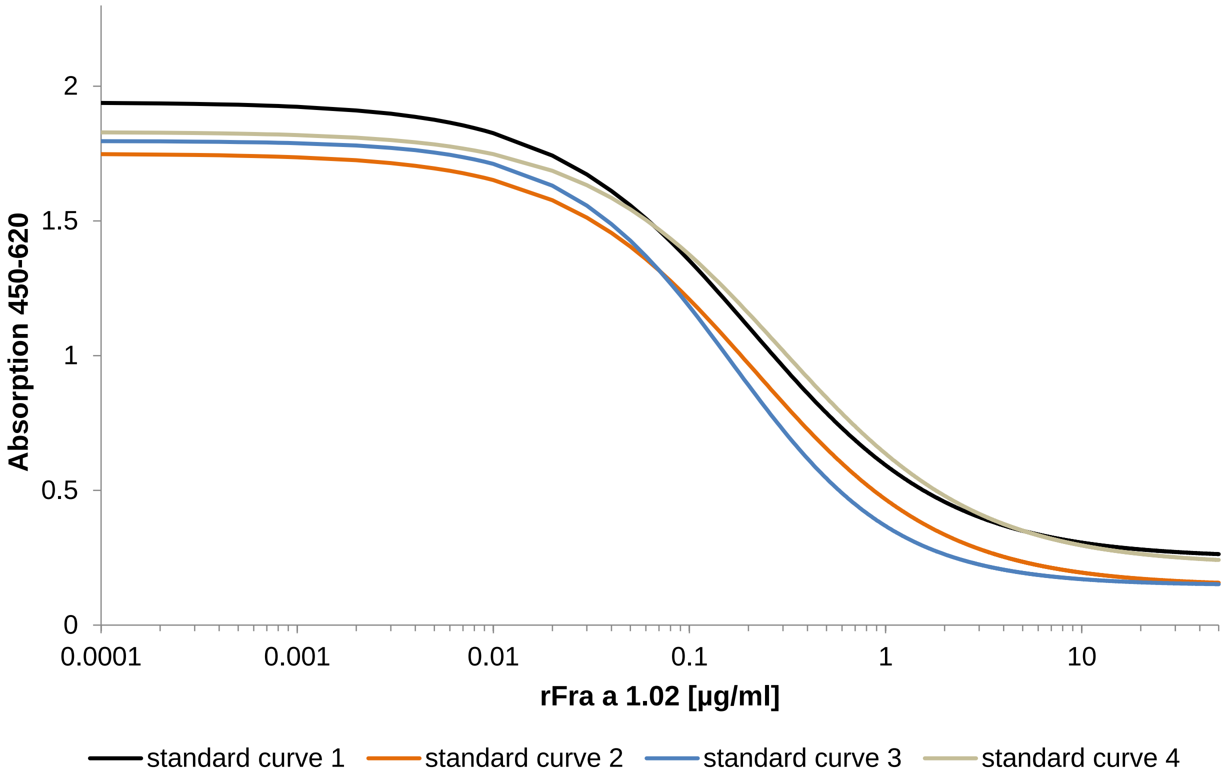

Figure S2. Standard curve of indirect competitive ELISA.

Competitive ELISA was performed using serial dilutions of rFra a 1.02 as free allergen. From the standard curves obtained at different days the concentration of Fra a 1 in µg/ml in various strawberry samples was calculated using the 4-paramter plot.

Table S1. Strawberry cultivars

Fra a 1 content (mean values) in µg/g FW, total soluble protein in µg/g FW and percentage of Fra a 1/total soluble protein of different strawberries. Plants were grown at Hansabred (Dresden, Munich).

variety	cultivar	color	Fra a 1 [µg/g FW]	total soluble protein [mg/g FW]	% Fra a 1/total soluble protein
F. × ananassa	Elianny	red	0.881 ± 0.91	1.014 ± 0.058	0.087
F. × ananassa	Elsanta	red	0.931 ± 0.27	1.002 ± 0.039	0.093
F. × ananassa	Faith	red	0.945 ± 0.31	0.888 ± 0.050	0.106
F. × ananassa	Königin Luise	red	1.025 ± 0.24	1.290 ± 0.104	0.079
F. × ananassa	Korona	red	1.185 ± 0.42	1.085 ± 0.064	0.109
F. × ananassa	Magnum	red	1.231 ± 0.28	1.168 ± 0.112	0.105
F. × ananassa	Mieze Schindler	red	0.985 ± 0.26	1.250 ± 0.065	0.079
F. × ananassa	Oberschlesien	red	0.957 ± 0.26	1.023 ± 0.077	0.094
F. × ananassa	Renaissance	red	0.968 ± 0.23	1.102 ± 0.043	0.088
F. × ananassa	Snow White	white	2.343 ± 0.75	0.981 ± 0.075	0.239
F. chiloensis	Lucida Perfecta	white	1.938 ± 0.66	1.359 ± 0.136	0.143
F. moschata	Profumata di Tortona	red	2.378 ± 1.02	1.441 ± 0.119	0.165
F. moschata	Wuerzburg	red	2.696 ± 0.79	1.590 ± 0.089	0.169
F. nilgerensis	Leigong	white	0.686 ± 0.13	1.722 ± 0.127	0.039
F. nilgerensis	Yunnan	white	1.339 ± 0.35	1.502 ± 0.163	0.089
F. vesca	Grotta del Vento	red	0.818 ± 0.24	1.794 ± 0.138	0.046
F. vesca	Moritzburg	yellow	0.937 ± 0.27	1.858 ± 0.104	0.050
F. vesca	Reine des Vallées	red	1.111 ± 0.43	2.044 ± 0.115	0.054
F. vesca	Yellow Wonder	yellow	1.331 ± 0.43	1.920 ± 0.062	0.069
F. × vesana	Florika	red	3.817 ± 1.28	1.385 ± 0.155	0.276

Table S2. Growing conditions for conventional and organic strawberry cv. Asia cultivated in Italy, Forlì-Cesena district.

	conventional	organic
**Name of Farm**	Integrated "Burioli Claudio"	Organic "Guardigni Paola"
**Location**	S. Martino in fiume (Cesena)	S. Martino in fiume (Cesena)
**Dimension**	8 hectares	10 hectares
**Age of the farm**	-	10 years
**Type of cultivation**	no-forced tunnel	semi-forced tunnel
**Plant transplant**	July	July
**Closing of tunnel**	May	March
**Planting spacing**	0.35 per 0.30 m	0.35 per 0.30 m
**Total production**	900 g/plant	850 g/plant
**Marketable production**	750 g/plant	700 g/plant
**Fertilization**	8-24-16 NPK, three times, 15 g/plant	Autumn cereal grass (*Triticum* spp.)
	Sequestrene, 0.05 g/plant	green manure at the end of April
		solid organic manure in June (before transplant)
		fertirrigation with products approved for Organic
**Treatments of pesticides**	commonly used anti-fungal and insecticides	Copper, Neem oil, Phytoseiids (against Spider mite)
**Harvest dates**	20 May 2015<br>24 May 2016<br>11 May 2017	20 May 2015<br>24 May 2016<br>11 May 2017
**Product destination**	cooperative and direct sale	direct sale

Table S3. Temperature and relative humidity at the growing location Forlì-Cesena in Italy for April and May in the years 2015 to 2017; National Agrometereological DataBase of Italy SIAN (National Agricultural Information System)

	Year	Month			
		**April**	**Historical Data (1960-1990)**	**May**	**Historical Data (1960-1990)**
**T_{min} [°C]**	2015	7.5		13.2	
	2016	9.0	8.2	11.6	12.2
	2017	7.6		12.1	
**T_{max} [°C]**	2015	20.7		26.5	
	2016	22.0	18.0	25.6	24.1
	2017	21.0		24.8	
**T_{mean} [°C]**	2015	14.1		19.8	
	2016	15.5	13.1	18.6	18.2
	2017	14.3		18.5	
**relative humidity [%]**	2015	85		69	
	2016	82		82	
	2017	81		71	
